# Examining validity and reliability of objective structured clinical examination for evaluation of clinical skills of midwifery undergraduate students: a descriptive study

**DOI:** 10.1186/s12909-020-02017-4

**Published:** 2020-03-31

**Authors:** Mitra Kolivand, Marzie Esfandyari, Sousan Heydarpour

**Affiliations:** 1 Department of Reproductive Health, Faculty of Nursing and Midwifery, Kermanshah University of Medical Sciences, Kermanshah, Iran; 2 Department of Midwifery, Faculty of Nursing and Midwifery, Kermanshah University of Medical Sciences, Kermanshah, Iran

**Keywords:** Clinical evaluation, Validity, Reliability, Midwifery

## Abstract

**Background:**

Clinical evaluation is one of the main pillars of medical education. The Objective Structured Clinical Examination is one of the commonly adopted practical tools to evaluate clinical and practical skills of medical students. The purpose of the study is to determine validity and reliability of Objective Structured Clinical Examination for evaluation of clinical skills of midwifery undergraduate students.

**Methods:**

Seven clinical skills were evaluated in this descriptive correlative study using a performance checklist. Census method was used for sampling. Thirty-two midwifery students performed the skills at seven stations each monitored by an observer using an evaluation checklist. Criterion validity was obtained through determining the correlation between the clinical and theoretical courses point and the Objective Structured Clinical Evaluation score. The collected data was analyzed in SPSS (v.20) and logistic regression test.

**Results:**

The correlation score of Objective Structured Clinical Examination was significantly related to the mean score of clinical course “Normal and Abnormal delivery I” (0.399, *p* = 0.024) and the mean score of clinical course “gynaecology “(0.419, *p* = 0.017). There was no significant correlation between OSCE scores and the mean score of theoretical courses (0.23, p = 0. 200).

The correlation between the total score and mean score of students at the stations showed that out of the seven stations, the correlations of the stations three (communication and collecting medical history) and four (childbirth) were not significant.

**Conclusion:**

Although, it appeared that Objective Structured Clinical Examination was one of the effective and efficient ways to evaluate clinical competencies and practical skills of students, the tool could not evaluate all the aspects.

## Background

Midwifery profession has a critical role in providing care to infants and mothers before and after childbirth [1]. A midwife should be equipped with critical thinking skills and making this happen needs a revolution in education and evaluation systems. About 50% of the midwifery education program is concentrated on clinical education [2], which is implemented as apprenticeship and internship courses. These courses play a key role in development of major and professional skills in midwifery students [2, 3].

Clinical evaluation methods and techniques to evaluate students’ skills and capabilities are highly important [4]. Evaluation of clinical competency in midwifery students is one of the toughest responsibilities of faculty board members and instructors of health programs [5, 6]. In addition, evaluation of clinical skills and competency in midwifery students is a challenge for education programs [7]. Different evaluation methods are needed to evaluated performance in nurses and midwives [8]. One of the most reliable and trusted methods to evaluate medical techniques is Objective Structured Clinical Examination (OSCE), which is mainly designed to evaluate clinical competency [9]. The point is that the traditional written and oral tests only measure the clinical knowledge while OSCE evaluates both the knowledge and skill [10]. In addition, the available clinical knowledge are not free from validity and reliability limitations. Structured practical methods can cover some of these limitations and OSCE is one of the most common structured practical tools [11].

The most important thing about implementation of OSCE test in midwifery and before internship is that this stage is a pivotal stage of practical trainings of midwives. At this stage, students are supposed to achieve the academic objectives of their program [12, 13]. The OSCE was first introduced by Harden and Dr. Glisone (1979) in Scotland to evaluate scientific information of students. Since then, the tools has been used in different countries [7, 14] and it has been established as one of the main methods of clinical competency assessment in medical education [15]. The well-designed and implemented OSCE can provide students with essential interpersonal, interviewing, problem-solving, and teaching assessment skills. The OSCE enables students to use basic clinical knowledge [16]. It empowers students to become active participants rather than the theory involved in coping with real-world situations. The OSCE engages students and enables them to understand the important factors involved in the nursing decision-making process. It also enables the students to perform advanced thinking and avoid errors in case-handling [17]. The OSCE, if implemented correctly, can have an effective role in completing the teaching-learning process and it can lead to an advanced training process [18]. The OSCE approach is an effective way of achieving educational goals that can reduce costs and promote clinical and practical education [19]. Moreover, throughout the test, the participants need to demonstrate specific clinical skills at different stations. Another feature of the test is that all students need to perform the same task at each station and there is a standard scoring system to evaluate the participants. The participants move from one station to another and deal with a specific clinical scenarios at each station [20]. The OSCE properly evaluates comprehensive capabilities and competencies so that the participants are given the chance to demonstrate different skills at different situations [11, 21].

The OSCE is currently used as a method to evaluate clinical skills and it has been adopted by Kermanshah School of Nursing and Midwifery since 2015. All midwifery students are evaluated using OSCE in terms of clinical competency before entering internship. Taking into account the importance of using valid tests to evaluate midwifery students, present study was conducted to determine the validity and reliability of OSCE for evaluation of clinical skills of midwifery undergraduate students before entering internship in 2018. The results of the study can be a step towards better evaluation methods in the education process.

## Methods

Study aim: To determine the validity and reliability of OSCE for evaluation of clinical skills of midwifery undergraduate students.

### Study setting and design

For developing the checklist, the issue was discussed at the department of midwifery, the faculty board members undertook to find women and midwifery-related references needed to design the stations of OSCE tests.

The deadline for brining in the references was 1 week. At the same time, the head of department and a member of faculty board visited the clinical skills training space of the school. The physical space and the available equipment were examined. The head of department coordinated the faculty board members to prepare a list of professional procedures and clinical performance needed in OSCE test to evaluate skills of the midwifery students. The deadline for preparing the list was 1 month. Then the head of department and other members of the faculty board determined the items of OSCE test. After that, the items were discussed at the department to determine the key stations of the test. Finally, seven stations, based on course topics, midwifery reference books and clinical skills requirements, including establishing open vein, serum therapy, measuring blood pressure; working with resuscitation equipment; communicating and collecting medical history skills; childbirth; episiotomy; speculum and bimanual exam, and visual questions was developed (Table 1). Overall the content of the test, the number of its stations, the time of each station (5 min), and the scoring system was decided by the faculty members of midwifery groups. Then checklists and a report of the objectives of study were provided to four experts and their feedbacks were used in the design of the final version of the test.

**Table 1:** Evaluated skills at different stations of OSCE test

Station	Evaluated skill
1	Establishing open vein, serum therapy, measuring blood pressure
2	Working with resuscitation equipment
3	Communication and collecting medical history skills
4	Childbirth
5	Episiotomy
6	Speculum and bimanual exam
7	Visual questions

Before the test, the head of department sent the list of 32 participants to the head of education affairs department to determine and notify the time and place of the test. The education department issued examination entrance card and delivered them to the students. In addition, the list of equipment needed for each station was given to the clinical skills education department. Five stations used Moulage, one used patient role playing, and one used a computer. The patient role was played by one of the experienced members of the faculty board.

The required scenario for each station was codified by the members of the board of faculty and affixed at the entrance of each partition. In addition, 80 visual question slides and 20 midwifery cases were prepared to examine the scientific level of the students. Two slides (displayed using a computer) and one written midwifery case were prepared for each student. After designing and preparing the stations, the OSCE was held for 1 day. In terms of ethics, an explanatory brief session was done 1 week before the exam by the head of department and the students were informed about the OSCE goals, the process, the time required for each station, and the order of attending each station. Moreover, a test guide pamphlet was given to the students.

In the exam day, the 32 students were grouped into four groups of seven members and one group of four members and the evaluation process was carried out. The students’ cellphones were collected before initiation of the test; then the students were allowed to enter the stations. To prevent students from exchanging viewpoints an examiner was in charge of controlling the students entering and leaving each station. In addition, the students were quarantined in two rooms before and after the test so that the students had no contact with each other.

Performance of each participant at each station was examined using the checklist of that stations by the instructors including researchers. After the exam, the students were given feedback. Then the checklists were collected and the students’ grades were determined for each station. The reliability of the assessors was calculated by determining the correlation between the scores reported by two observers at the station “ Communication and collecting medical history skills”, due to the subjective of this station.

### Sampling techniques

Census method was used for sampling. This methodological study was done on 32 undergraduate midwifery students in the 6th semester at the Nursing and Midwifery School of Kermanshah University of Medical Sciences in 2018. Inclusion criteria were being in pre-internshipstage, passing all theoretical and clinical courses, and no experience with the OSCE test.

### Data collection

The data collection tool was a clinical skill checklist, including seven procedures (seven stations) (Table 1). Each procedures constituted of several practical skills. The checklists covered the procedures step by step and each step had a specific score. The questions of checklists were scored as failed (0), low (1), moderate (2), good (3), and excellent (4). The procedure, “diagnosis and knowledge” were scored as failed/successful diagnosis or correct/wrong answer. The score for each station was calculated by dividing the total obtained score by the number of items in the related checklist and the total score was the sum of scores of all the seven stations.

### Data analysis

The Kolmogorov Smirnov (KS) test was used to determine the normality of the data. The data were analyzed using Pearson correlation test, Spearman Correlation test and Cronbach’s alpha test in SPSS (v.16) (*P* = 0.05).

## Results

Data analysis showed that the mean age of the students was 22.78 ± 1.40 and the age range was 21–26 years. Moreover, 22.2% of the participants were unmarried. Total mean scores of theoretical courses was 15.98 ± 1.07. The evaluated skills based on different stations are listed in Table 1.

The face validity of the checklist was confirmed using the clinical experience of the instructors of practical skills. Content validity was approved by providing the content of the test to the faculty members of midwifery groups and using feedbacks by four experts about the Content.

To determine the criterion validity, the mean scores of clinical courses recorded in the students’ files was used and the correlation between these scores and total OSCE score was obtained.

In addition, the correlation of the mean of theoretical courses with the total score of OSCE was taken into account.

The results indicated that the correlation of OSCE scores with the mean score of clinical course “Normal Pregnancy I”, the mean score of clinical course “Normal and Abnormal delivery I”, and the mean score of clinical course “gynaecology” was 0.319 (*p* = 0.075), 0.399 (*p* = 0.024), and 0.419 (*p* = 0.017) respectively. Moreover, there was no significant correlation between OSCE scores with the mean score of theoretical courses (Table 2).

**Table 2:** Correlation between the scores of clinical courses, “Normal and Abnormal delivery I, Normal Pregnancy I, gynecology and total average scores and OSCE score

Variable	Total score		
	Mean ± SD	R	*P*-value
clinical course “Normal Pregnancy I”	16.46 ± 0.26	0.319	0.075
clinical course “Normal and Abnormal delivery I”	16.13 ± 0.60	0.399	0.024
clinical course “gynecology	17.86 ± 0.33	0.419	0.017
“total average scores”	07/1 ± 98/15	0.23	0.200
Total OSCE score	11.53 ± 1.74	1	–

To determine internal consistency, the correlation of the total scores of the test and the scores of each station was obtained. The Pearson Correlation test showed that there was a significant correlation between OSCE score (total score) and the scores of stations 1, 2, 5, 6, and 7 (*p* < 0.05). In addition, the Spearman Correlation test showed no significant relationship between OSCE score (total score) and the scores of stations 3 and 4 (*p* > 0.05) (Table 3). Finally, to determine reliability of the test, Cronbach’s alpha was calculated with an emphasis on internal consistency. The results of the assessors’ reliability and the correlation between the observers’ scores in the station “ Communication and collecting medical history skills “, showed that the agreement between the observers of assessor was desirable(*P* < 0.001 r = 0.)

**Table 3:** Correlation coefficients between the scores of different stations and the total score

Variable	Total score		
	Mean ± SD	R	*P*-value
Station 1	1.24 ± 0.41	0.726	0.001
Station 2	1.29 ± 0.34	0.394	0.026
Station 3	1.54 ± 1.02	0.340	0.057
Station 4	1.47 ± 0.26	0.291	0.106
Station 5	1.92 ± 0.16	0.387	0.029
Station 6	1.97 ± 0.26	0.501	0.003
Station 7	2.57 ± 1.06	0.805	0.001
Total score	11.53 ± 1.74	1	–

## Discussion

The validity and reliability of the OSCE held for pre-internship midwifery students at Kermanshah University of Medical Sciences in 2018 were supported. This finding is consistent with other studies in medical fields [8, 22, 23]. Villegas et al. (2016) showed that OSCE was a reliable method to evaluate clinical competency and they recommended using it as a part of educational curriculum [24]. Michael and Villegas (2014) used OSCE test to prepare undergraduate midwifery students in Australia using stations like examining C-section infants, and post-delivery monitoring. Based on the midwifery students’ comments, OSCE had a positive effect on their self-confidence and performance [25]. Smith et al. (2012), showed in a review study that clinical competency of nursing and midwifery students was improved using OSCE test for skills like midwifery emergencies, pharmacology, drug prescription, breastfeeding, and supplementary nutrition in infants. They argued that the test was a valuable strategy to evaluate clinical competency and improving knowledge of students. In addition, the test was a motivating factor to create diversity in midwifery students’ education [26]. Omu [26], stated that OSCE test was a golden standard evaluation method and it was recommended for the evaluation of clinical competency and psychomotor skills [27]. Despite differences between the mentioned studies in terms of the skills under study, their results about the strengths and weaknesses are consistent.

According to our results, face validity was appropriate, which was consistent with other studies in this field [28, 29].

In this study, content validity was approved by providing the content of the test to the faculty members of midwifery department and using feedbacks of four experts about the content and design of the final version of the test. Setyonugroho et al.(2016) believed that skill assessment checklists in objective structured tests and the development of comprehensive scenarios according to the station’s context represent the content validity of the test’ [30].

Criterion validity results showed a positive and significant relationship between the total score of OSCE and clinical courses (clinical course “Normal and Abnormal delivery I” and clinical course “gynaecology). Therefore, the criterion validity was desirable and consistent with our study, Moatari et al. (2007) showed that there was a significant and positive relationship between OSCE scores and nursing clinical courses scores [8]. Moreover, Farajzadeh et al. (2012) used stepwise multivariate regression test to determine predictability OSCE score using clinical and theoretical courses scores. The results showed that the clinical courses scores can be used to determine OSCE score, while there was no relationship between theoretical courses scores and OSCE score [4]. A positive and significant relationship between the clinical courses and the OSCE is expected so that the simulated environment can be combined with actual environment to enhance student learning. Contrary to our results, Sabzi et al. (2018) found a low correlation between the mean score of clinical courses and the OSCE score. They stated that this may be due to the difference between the real and the simulated environments, because working with a real patient gives people more motivation than the simulated environments. On the other hand, usually general assessment sheets are used to assess students in clinical field environments; while in the OSCE each procedure is assessed separately and in full detail [28].

In addition, our results showed no significant correlation between OSCE scores and the mean score of theoretical courses. Consistent with our results, Raj et al. (2007) reported no significant correlation between Rheumatology written exam score (hand section) and total OSCE score (22). Moatari et al. (2007) also reported that there was a positive and weak relationship between OSCE scores and nursing theoretical courses scores [8]. According to Gray (2010) students might possess a wide theoretical knowledge and good theoretical scores; however, this does not necessarily translated into good performance in the clinical environment’ [31]. The gap between theoretical and practical courses has always been an educational challenge, and the low correlation between OSCE test and theoretical courses supports existence of this gap [8]. Alami and Safari (2014) proposed not to design stations in OSCE test based on written scenarios and recommended using stations based on patient role play, moulage, and mannequin. The reason for these recommendations is that using written scenarios concentrate the tests on the sections that are measured by pre-apprenticeship test [32]. Contrary to results of present study, Gerrow et al. (2003) tried to examine criterion validity of OSCE test through evaluating the dentistry practical skills based on written board exam scores of 2317 senior students in 5 years. They showed a positive and significant relationship between the written exam scores and OSCE results [33]. Sabzi et al. (2018), found the higher correlation between theoretical score and OSCE score compared to clinical score and OSCE score [28]. Moattari (2013) argued that in spite of all the works on objective tests in the fields of knowledge, skills, and attitudes, theoretical tests are better in measuring the qualifications and competence of individuals [34].

To examine internal consistency, the correlation between total OSCE test and mean score of each student at each station was computed. The results showed that out of seven stations, the correlation coefficient was not significant for two stations (No. 3 & 4). In this way, internal consistency of the test is approved, which is consistent with the studies of other researchers [28, 29].

The significant correlation between the majority of stations and total score of OSCE indicates that the participants gained the required skills during the pre-internship courses. Hosseini et al. (2013) reported that out of ten stations, correlation coefficients of two stations (8 and 10) were not significant [23]. Taghva et al. (2007), studied psychology skills and reported that out of nine stations, correlation coefficient of only one was insignificant [35]. Moreover, Moatari et al. (2009), evaluated nursing skills and reported that out of 10 stations, only two had low internal consistency [9]. Differences between these studies can be explained based on the design of stations, the way of holding briefing sessions of OSCE test, and complicacy of the instructions. Wilkinson et al. (2000) and Tudiver et al. (2009) stated that the correlation between the skill stations scores and the total score indicates the construct validity [36, 37].

The reliability of the OSCE and the total score of Cronbach’s alpha supported internal consistency (α > 0.7), which was consistent with other studies [23, 38–40]. Bould et al. (2009), reported a Cronbach’s alpha coefficient in 0.8 and 1 range, which indicates a good reliability of the tool under study [41]. Metsamuronen et al. (2006) reported that the minimum acceptable Cronbach’s alpha was 0.7 [42]. Specified criteria for skill scoring and training of the students were some of reliability factors of the tests, which were used in the present study.

### Study limitation

As to limitations of the study, tiredness of the evaluators and the students’ concerns about their probable negative impression on instructors due to their poor performance are notable. The number of students was small (*n* = 32) which makes the statistics ‘unstable.’ Considering the low R^2^, the explaining variability of the correlations presented questionable and can’t be changed due to the limited number of students in the studied cohort. The reliability of the tool was not examined in this study, and future studies may consider examining the reliability of the tool. Moreover, future studies may implement OSCE test in different occasions including immediately, 1 day, 72 h, and 2 months after later in another study.

## Conclusion

Although, it seems that OSCE test is an effective and efficient way to measure clinical competencies and practical skills of students, it did not cover all the aspects. Effectiveness of the tool depends on factors like experienced evaluators, access to references and equipment, adequate time to design and implement the test, accurate planning, availability of a proper space to hold the test, and suitable measurement tools.

## Data Availability

The datasets used and/or analyzed during the current study can be provided by the first author on a reasonable request.

## Abbreviations

OSCE: Objective Structured Clinical Examination
